# Associations between Adherence to the Taiwan Dietary Reference Intakes of Micronutrients and the Risk of Type 2 Diabetes

**DOI:** 10.3390/ijerph191912242

**Published:** 2022-09-27

**Authors:** Ming-Chieh Li

**Affiliations:** Department of Health Promotion and Health Education, College of Education, National Taiwan Normal University, Taipei City 106, Taiwan; mingchiehli@ntnu.edu.tw; Tel.: +886-277491698

**Keywords:** diabetes, vitamin A, vitamin B1, dietary reference intakes (DRIs), micronutrient

## Abstract

The dietary reference intakes (DRIs) were established as guidance for the intake of micronutrients and other nutrients. However, how DRIs affect disease status has not been thoroughly examined. The aim of this study is to examine the associations between adherence to the DRIs of selected micronutrients and type 2 diabetes. A populational and cross-sectional study was conducted using data from the Nutrition and Health Survey in Taiwan (NAHSIT) 2013–2016. A total of 14 nutrient intakes, including those for vitamin A, C, D, E, B1, B2, B3, B6, B12, iron, magnesium, zinc, calcium, and phosphorus, were evaluated using the 24 h dietary recall method. Type 2 diabetes was defined as a fasting serum HbA1c level of 6.5% or higher and excluded participants who had received treatments for type 2 diabetes or reported a diagnosis of type 2 diabetes by physicians to avoid the possible inverse association. A total of 2685 participants aged 19 and above were included in the final analysis. After adjusting for confounders, we found that adherence to the DRI of vitamin A was associated with a reduced risk of type 2 diabetes among men. The odds ratio (OR) was 0.64 (95% confidence interval (CI) = 0.42–0.99) compared with men who did not adhere to the DRI. As for women, we found that adherence to the DRI of vitamin B1 was associated with a reduced risk of type 2 diabetes. The OR was 0.59 (95% CI = 0.35–0.97) compared with that for women who did not adhere to the DRI. This study showed that adherence to Taiwan DRIs of vitamin A for men and vitamin B1 for women might have beneficial effects on type 2 diabetes.

## 1. Introduction

The prevalence and global burdens of type 2 diabetes continue to increase around the world, especially in developed countries [1]. An estimated 536.6 million adults were living with diabetes worldwide in 2021, and this will increase to 783.2 million in 2045 [2]. Among them, an estimated one-third of diabetes-related deaths occur in people under the age of 60 [3], which not only induces premature mortality [4] but also creates a heavy economic burden on society [5]. Due to the effects caused by type 2 diabetes, academics and policymakers need to identify prevention strategies.

Type 2 diabetes might be induced through several possible mechanisms, including micronutrient status [6,7,8]. For example, zinc and chromium have been found to be involved in the pathogenesis of glucose intolerance [9], and increased chromium intakes might be associated with a lower risk of type 2 diabetes. A systematic review and meta-analysis concluded that zinc supplementation has beneficial effects on glycemic control [10]. Antioxidant micronutrients also play a role in the development of type 2 diabetes. For instance, studies have indicated that low levels of vitamin E might be a risk factor for type 2 diabetes. Some clinical trials have suggested a protective effect of vitamin E supplementation on glycemic control [11,12]. A recent study found that among diabetic patients, 58.7% had vitamin C deficiency [13]. 

Many governments and policymakers have published dietary reference intakes (DRIs) as guidance for the intakes of micronutrients and other nutrients. This is the case in Taiwan. However, although previous studies have suggested that micronutrient status are associated with the risk of type 2 diabetes, how adherence to the DRIs of micronutrients associated with the risk of type 2 diabetes is not clear. We therefore conducted a study to examine the associations between adherence to the DRIs of selected micronutrients and the risk of type 2 diabetes in the Taiwan general population.

## 2. Materials and Methods

### 2.1. Study Design and Participants

We used a dataset obtained from the Nutrition and Health Survey in Taiwan (NAHSIT) 2013–2016, which was a populational representative survey in Taiwan monitoring the Taiwanese nutritional status. The survey method is described elsewhere [14]. The NAHSIT collected demographic data through face-to-face interviews. Participants were also invited to attend a physical examination at a temporary health examination station.

The diagram of the sample selection process is shown in Supplemental Figure S1. A total of 11,072 participants aged two months and above were included in the NAHSIT survey. The present study restricted participants to age 19 and above according to the definition of adults adapted from the newest version of DRIs (8th edition) (*n* = 5770). Among them, 3075 participants had received a physical examination and had complete demographic and 24 h dietary recall data. Participants without data on HbA1c levels were excluded (*n* = 110). Among 2965 participants with data on HbA1c levels, we further excluded participants who received treatments for type 2 diabetes or reported a diagnosis of type 2 diabetes by physicians to avoid the possible inverse associations. Finally, a total of 2685 participants were included in the final analysis. This study was conducted in accordance with the principles of the Declaration of Helsinki and was approved by the China Medical University and Hospital Research Ethics Center (CRREC-108-136). All participants have signed informed consent during the Nutrition and Health Survey, including consent for their data to be used for future research purposes.

### 2.2. Definition of Type 2 Diabetes

Type 2 diabetes was defined as a fasting serum HbA1c level of 6.5% or higher. Type 2 diabetes patients might modify their dietary behavior and result in changes of dietary intakes. To avoid the possible inverse associations, we excluded participants who had received treatments for type 2 diabetes or who reported a diagnosis of type 2 diabetes by physicians.

### 2.3. Assessments of the Micronutrient Adequacy

The trained interviewers from the NAHIST conducted door-to-door interviews to collect 24 h dietary recall data among all participants [15]. To reduce the intra-individual variance, the NAHSIT randomly selected participants among different age groups and conducted a repeated 24 h recall survey one week after the first survey. The results of repeated surveys were used for the adjustment of the 24 h recall data. The detailed methods can be found elsewhere [16].

In the NAHSIT, a total of fourteen nutrient intakes, including those for vitamins A, C, D, E, B1, B2, B3, B6, B12, iron, magnesium, zinc, calcium, and phosphorus, were evaluated. We examined the associations between adherence to the DRIs of selected micronutrients (8th edition in Taiwan) and the risk of having type 2 diabetes. The DRI adherence was estimated by the prevalence of participants whose intake was lower than the Recommended Dietary Allowance (RDA) or Adequate Intakes (AI) for selected micronutrients. The DRIs used in this study are shown in Supplemental Table S1, which was made by the authors according to the newest version of DRIs in Taiwan (https://www.hpa.gov.tw/Pages/List.aspx?nodeid=4247, accessed on 1 August 2022). 

### 2.4. Statistical Analysis

The associations of baseline personal characteristics with type 2 diabetes were evaluated using Chi-square or Fisher’s exact tests. We applied the logistic regression models to determine whether adherence to the DRIs of selected nutrients was associated with risk of type 2 diabetes. Potential confounding factors were selected based on prior knowledge and their relations with exposures and outcomes. Stratified analyses were conducted by sex. All analyses were performed using SAS 9.4 (SAS Institute, Cary, NC, USA).

## 3. Results

Supplemental Table S1 displays the sex-specific Taiwan DRIs of the selected 14 micronutrients. Table 1 shows the demographic comparisons between diabetic (HbA1c > 6.5%) and non-diabetic participants. A total of 2685 participants aged 19 and above were included in the final analysis. Compared with participants without diabetes, diabetic patients were more likely to be men (57.23% vs. 47.53%); to be older (age ≥ 71: 45.09% vs. 25.40%); to have a lower education level (college or above: 10.98% vs. 23.96%); to be divorced, separated, widowed, or refuse to answer (24.86% vs. 13.69%); to have lower family income (family income ≥ NT $80,000: 19.08% vs. 24.48%); and to have lower physical activity (median or high physical activity: 47.40% vs. 64.65%).

Among men, after adjusting for age, body mass index, education level, marital status, family income, and physical activity, we found that adherence to the DRI of vitamin A was negatively associated with risk of type 2 diabetes. The odds ratio (OR) was 0.64 (95% confidence interval (CI) = 0.42–0.99) compared with those who did not adhere to the DRI (Table 2). We found no significant association between the other micronutrients and the risk of having diabetes.

Among women, after adjusting for confounding factors, we found that adherence to the DRI of vitamin B1 was associated with a reduced risk of type 2 diabetes. The OR was 0.59 (95% CI = 0.35–0.97) compared with those who did not adhere to the DRI (Table 3). We found no association between the other micronutrients and the risk of having diabetes.

## 4. Discussion

In the present study, we observed sex-specific effects between adherence to the DRIs of vitamin A and vitamin B1 and the risk of type 2 diabetes. Protective effects were found between adherence to the DRIs of vitamin A and risk of type 2 diabetes, although the finding regarding women did not reach statistical significance. In contrast, the effects of vitamin B1 showed different effect directions. Among women, adherence to the DRIs of vitamin B1 was negatively associated with type 2 diabetes, whereas adherence to the DRI of vitamin B1 showed a slightly increased but non-significant risk of having type 2 diabetes among men. 

To the best of our knowledge, there was only one study conducted in a Japan hospital investigating the dietary intakes of individuals with type 2 diabetes and characterizing their intakes relative to the DRIs [17]. They found that dietary inadequacy relating to vitamins and minerals was greater in men than in women. However, the study only presented the percentages of type 2 diabetic patients who have intakes less than the estimated average requirement (EAR) or AI. There was no control group in this study. This approach examined the nutritional status among type 2 diabetic patients, but not how adherence to DRIs affected the risk of type 2 diabetes. In the present study, in contrast, we examined how adherence to the DRIs of micronutrients associated with the risk of type 2 diabetes. In addition, to avoid a possible inverse relationship, we excluded participants who knew they had type 2 diabetes. Our findings might be a reference for preventing the risk of type 2 diabetes.

Vitamin A has been associated with both type 1 and type 2 diabetes. Studies have shown that type 1 diabetes leads to a reduction in blood concentrations of vitamin A [18,19]. On the other hand, mutual and mixed effects were found between blood concentrations of vitamin A and type 2 diabetes. A case–control study showed that the presence of diabetes was not associated with blood concentrations of vitamin A [20]. Two studies conducted in South Korea and the United Kingdom found that impaired fasting glucose or glucose tolerance was associated with higher blood vitamin A levels [21,22]. Some studies identified an increased risk of type 2 diabetes among subjects with higher serum vitamin A or provitamin A (β-carotene) [23,24]. A populational-based study in South Korea suggested that serum retinol was associated with higher fasting glucose; however, the effect was not significant after adjusting for BMI [25]. Studies of the dietary vitamin A intake on type 2 diabetes are relatively limited. One study has indicated that increased provitamin A (β-carotene) intake was associated with a reduced risk of type 2 diabetes [26]. Another study of South Asians in the United States found that type 2 diabetic patients had a lower intake of vitamin A than controls [27]. However, a study among 19,168 healthy Japanese found no association of vitamin A with the incidence of type 2 diabetes [28]. The present study added evidence that higher vitamin intake is associated with a lower risk of type 2 diabetes among men. Although previous studies have found associations between concentrations of vitamin A and type 2 diabetes, this might be a consequence of depleted or altered carbohydrate intolerance and insulin resistance [6].

Vitamin B1, also known as thiamine, has been associated with the risk of type 2 diabetes. Studies have found that diabetic patients reported lower concentrations of vitamin B1 [29], which might be due to increased urinary thiamine excretion. As for vitamin intake from diet or supplement, randomized controlled trials suggested that thiamine supplements might improve therapy for early-stage diabetic nephropathy [30] and are needed for diabetic patients who enrolled in the weight loss program [31]. A study conducted in Korea found that dietary intake of vitamin B1 was associated with a reduced risk of type 2 diabetes [32], which was consistent with our findings in women. One study assessed the associations between daily total dietary nutrient intake and glycemic control states using the National Health and Nutrition Examination Survey (NHANES) data [33]. The NHANES was the USA general population examination survey. The authors found that the intake of vitamin B1 was associated with better glycemic control (HbA1c < 6.5%) after adjusting for gender, age, and race, which was consistent with our findings. However, after further adjusting for the other confounding factors, the effect disappeared. The reason for this might be due to overadjustment or collinearity across the included covariates [34]. The authors have adjusted all the models for some possible intermediate variables, such as hypertension status, which might cause biased findings. Moreover, the authors have adjusted all the models for insulin and glucose levels, which were considered to be highly correlated with HbA1c levels. It might result in collinearity and potential biased findings. The evidence regarding vitamin B1 is relatively limited, especially since large-scale RCTs and cohort studies to confirm the role of vitamin B1 in diabetes are lacking. More RCTs are needed to obtain robust conclusions.

The current study has some limitations. The most important limitation of this study was the cross-sectional design, meaning that causality cannot be established. However, we have excluded participants who had received treatments for type 2 diabetes or those who had self-reported physician-diagnosed type 2 diabetes so that the possibility of obtaining an inverse relationship would be minimized. The one-time assessment of dietary intake and HbA1c level might have led to misclassification. However, non-differential misclassification usually yields measures of association that are closer to null, suggesting that the observed findings might be underestimated. The intra-individual variance was considered the major limitation of using the 24 h dietary recall method as the dietary assessment tool. However, NAHSIT performed repeated surveys for adjustment of the 24 h recall data. Therefore, the potential bias could be minimized. Finally, in this study, only 14 micronutrient intakes were included in the data analyses. The effects of other important micronutrients, such as selenium and copper, were not examined due to a lack of data. More studies are needed to explore their effects on type 2 diabetes.

The strengths of this study included its population-based study design, which might allow us to generalize the findings to the public. The fair sample size allowed us to detect possible associations between micronutrient intakes and type 2 diabetes. In addition, unlike most previous studies, we examined the association between adherence to DRIs, not merely the intake of micronutrients, and the risk of type 2 diabetes. This kind of approach has clinical significance and could be a reference for policymakers. 

## 5. Conclusions

In conclusion, our study showed that adherence to the Taiwan DRIs of vitamin A for men and vitamin B1 for women might have beneficial effects on type 2 diabetes. However, more cohort studies are warranted to confirm the observed findings. 

## Figures and Tables

**Table 1:** Demographic descriptions of included study participants (*n* = 2685).

	HbA1c ≤ 6.5%	HbA1c > 6.5%	
Variables	*n* = 2512	*n* = 173	*p*-Value
Sex			
Men	1194 (47.53%)	99 (57.23%)	0.01
Women	1318 (52.47%)	74 (42.77%)	
Age			
19 ≤ age ≤ 30	745 (29.66%)	10 (5.78%)	<0.0001
31 ≤ age ≤ 50	379 (15.09%)	18 (10.40%)	
51 ≤ age ≤ 70	750 (29.86%)	67 (38.73%)	
Age ≥ 71	638 (25.40%)	78 (45.09%)	
Body mass index			
BMI < 24	1392 (55.41%)	36 (20.81%)	
27 > BMI ≥ 24	606 (24.12%)	60 (34.68%)	
BMI ≥ 27	514 (20.46%)	77 (44.51%)	
Education			
Elementary school	1300 (51.75%)	81 (46.82%)	<0.0001
Junior high and high school	610 (24.28%)	73 (42.20%)	
College or above	602 (23.96%)	19 (10.98%)	
Marital status			
Single	480 (19.11%)	8 (4.62%)	<0.0001
Married or lived together	1688 (67.20%)	122 (70.52%)	
Divorced, separated, widowed, or refused to answer	344 (13.69%)	43 (24.86%)	
Family income			
Income < NT $10,000	183 (7.29%)	23 (13.29%)	<0.01
NT $10,000 ≤ income < NT $40,000	498 (19.82%)	37 (21.39%)	
NT $40,000 ≤ income < NT $80,000	641 (25.52%)	33 (19.08%)	
Income ≥ NT $80,000	615 (24.48%)	33 (19.08%)	
Do not know or refuse to answer	575 (22.89%)	47 (27.17%)	
Physical activity			
Low	888 (35.35%)	91 (52.60%)	<0.0001
Median or high	1624 (64.65%)	82 (47.40%)	

NT: The New Taiwan dollars; NT $10,000 = $324.30 USD; NT $40,000 = $1297.19 USD; NT $80,000 = 2594.37 USD; BMI: body mass index; HbA1c: Hemoglobin A1c.

**Table 2:** The associations between adherence to the DRIs of selected micronutrients and the risk of having type 2 diabetes (men).

	HbA1c < 6.5%	HbA1c ≥ 6.5%
Nutrient intakes		
Vitamin A	1	0.64 (0.42–0.99)
Vitamin C	1	1.08 (0.70–1.67)
Vitamin D	1	1.08 (0.60–1.96)
Vitamin E	1	1.10 (0.66–1.84)
Vitamin B1	1	1.15 (0.74–1.79)
Vitamin B2	1	0.81 (0.51–1.27)
Vitamin B6	1	0.73 (0.47–1.14)
Vitamin B12	1	1.13 (0.72–1.77)
Vitamin B3	1	0.99 (0.63–1.54)
Iron	1	0.70 (0.43–1.14)
Magnesium	1	0.70 (0.44–1.11)
Zinc	1	1.01 (0.64–1.58)
Calcium	1	0.68 (0.34–1.37)
Phosphorus	1	1.07 (0.61–1.85)

HbA1c: Hemoglobin A1c; DRIs: Dietary Reference Intakes. Exposure variable: DRI adherence of selected micronutrients. Outcome variable: the risk of type 2 diabetes (HbA1c ≥ 6.5%). All the logistic regression models were adjusted for age, body mass index, education level, marital status, family income, and physical activity.

**Table 3:** The associations between adherence to the DRIs of selected micronutrients and the risk of having type 2 diabetes (women).

	HbA1c < 6.5%	HbA1c ≥ 6.5%
Nutrient intakes		
Vitamin A	1	0.69 (0.42–1.13)
Vitamin C	1	0.71 (0.43–1.17)
Vitamin D	1	1.16 (0.54–2.48)
Vitamin E	1	0.72 (0.32–1.65)
Vitamin B1	1	0.59 (0.35–0.97)
Vitamin B2	1	0.82 (0.49–1.36)
Vitamin B6	1	0.88 (0.53–1.46)
Vitamin B12	1	1.09 (0.66–1.81)
Vitamin B3	1	0.99 (0.60–1.65)
Iron	1	0.92 (0.55–1.54)
Magnesium	1	0.89 (0.52–1.53)
Zinc	1	0.69 (0.37–1.30)
Calcium	1	0.49 (0.17–1.40)
Phosphorus	1	0.89 (0.53–1.50)

HbA1c: Hemoglobin A1c; DRIs: Dietary Reference Intakes. Exposure variable: DRI adherence of selected micronutrients. Outcome variable: the risk of type 2 diabetes (HbA1c ≥ 6.5%). All the logistic regression models were adjusted for age, body mass index, education level, marital status, family income, and physical activity.

## Data Availability

Data from the Nutrition and Health Survey in Taiwan (NAHSIT) are available for research purposes, but an application must be submitted to, and approved by the Ministry of Health and Welfare in Taiwan.

## Supplementary Materials

The following supporting information can be downloaded at: https://www.mdpi.com/article/10.3390/ijerph191912242/s1. Table S1. Taiwan Dietary Reference Intakes of selected micronutrients. Figure S1. Diagram of the sample selection process for this study (*n* = 2685).
