# Supplementary material for: Associations between Adherence to the Taiwan Dietary Reference Intakes of Micronutrients and the Risk of Type 2 Diabetes
Source: Int J Environ Res Public Health. 2022 Sep 27;19(19):12242. doi: 10.3390/ijerph191912242 (PMC9566350; doi:10.3390/ijerph191912242)
Supplement: Supplementary file 1 [file ijerph-19-12242-s001.zip › ijerph-1897994-supplementary.pdf]

Table S1. Taiwan Dietary Reference Intakes of selected micronutrients

Nutrients	Dietary Reference Intakes	Reference
Vitamin A	Aged 19 and above: 600 μg RE for men, 500 μg RE for women.	RDA
Vitamin C	Aged 19 and above: 100 mg.	RDA
Vitamin D	Aged 19 to 50: 10 μg;<br>Aged 51 and above: 15 μg.	AI
Vitamin E	Aged 19 and above: 12 mg α-TE.	AI
Vitamin B1	Aged 19 and above: 1.2 mg for men, 0.9 mg for women.	RDA
Vitamin B2	Aged 19 and above: 1.3 mg for men, 1.0 mg for women.	RDA
Vitamin B3	Aged 19 and above: 16 mg NE for men, 14 mg NE for women.	RDA
Vitamin B6	Aged 19 to 50: 1.5 mg;<br>Aged 51 and above: 1.6 mg.	RDA
Vitamin B12	Aged 19 and above: 2.4 μg.	RDA
Iron	Aged 19 to 50: 10 mg for men, 15 mg for women;<br>Aged 51 and above: 10 mg.	RDA
Magnesium	Aged 19 to 50: 380 mg for men, 320 mg for women;<br>Aged 51 to 70: 360 mg for men, 310 mg for women;<br>Aged 71 and above: 350 mg for men, 300 mg for women.	RDA
Zinc	Aged 19 and above: 15 mg for men, 12 mg for women.	AI
Calcium	Aged 19 and above: 1000 mg.	AI
Phosphorus	Aged 19 and above: 800 mg.	AI

RE: retinol equivalent; α-TE: α-tocopherol equivalent; NE: niacin equivalent; RDA: recommended dietary allowance; AI: adequate intakes.

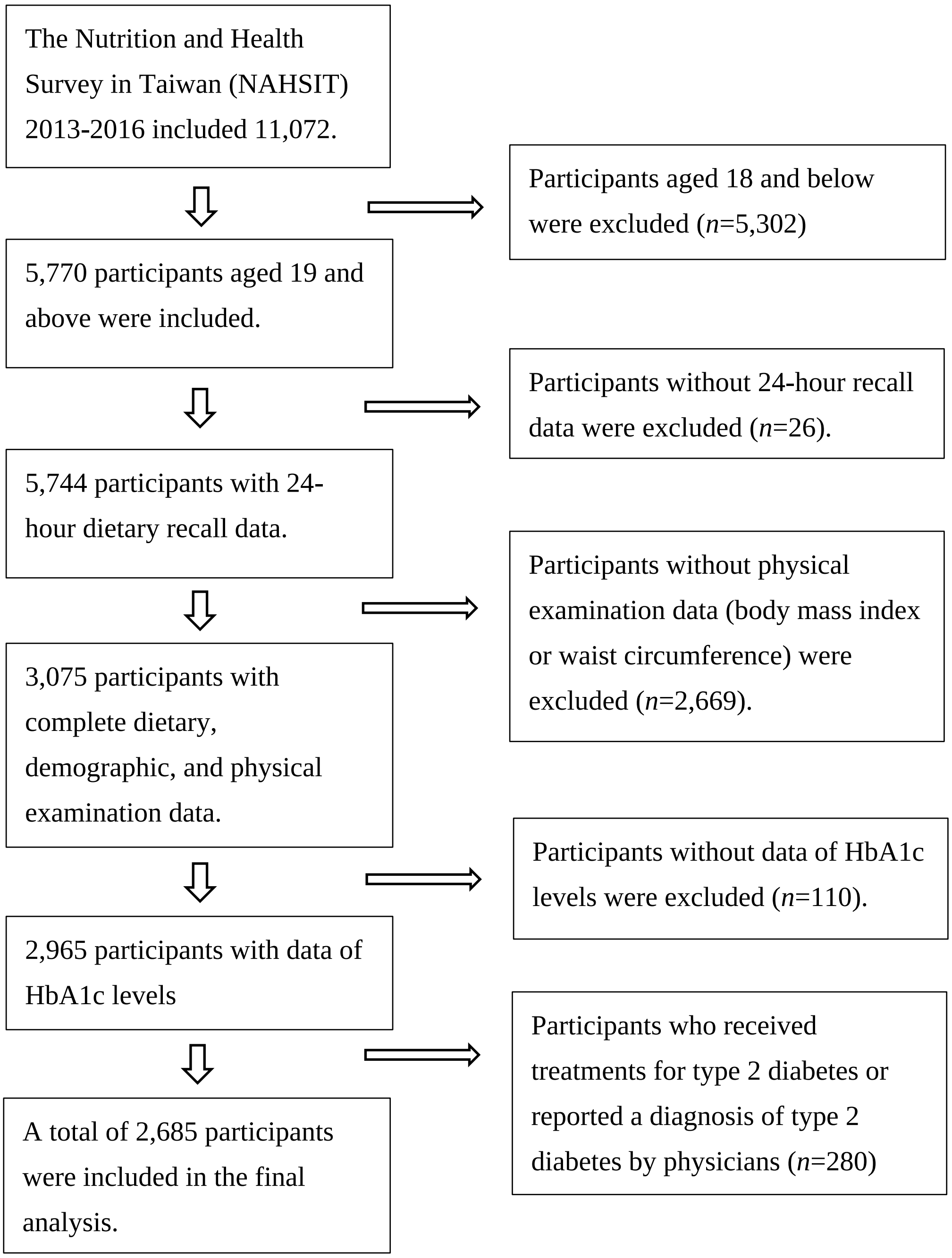

Figure S1. Diagram of the sample selection process for this study (*n*=2,685).
